# Editorial: The neural circuitry of mating behaviors

**DOI:** 10.3389/fncir.2022.1102051

**Published:** 2023-01-06

**Authors:** Stefano Zucca, Adam C. Puche, Serena Bovetti

**Affiliations:** ^1^Department of Life Sciences and Systems Biology, University of Turin, Turin, Italy; ^2^Neuroscience Institute Cavalieri Ottolenghi, University of Turin, Turin, Italy; ^3^Department of Anatomy and Neurobiology, University of Maryland School of Medicine, Baltimore, MA, United States

**Keywords:** mate choice, sensory integration, multimodal communication, sexual signals, sensory systems

The ability to choose the most suitable reproductive partner is one of the primary drivers of evolution. The process of mate attraction and selection strongly relies on multimodal communication, where stimuli within a species typically originate from at least two different sensory modalities (Bastock, 1967). Despite many studies focused on understanding the ethological role of multimodal communication during mating, very little is known about the neuronal mechanisms involved in the integration of multisensory sexual cues. This editorial gathered contributions from experts in the field of mating behaviors in multiple animal species to build a more comprehensive and comparative understanding of multisensory processing during mate selection and their underlying neuronal circuits.

## Multimodal communication during courtship

Courtship is defined as the behavior used to obtain or maintain reproductive interactions with a partner (Skinner, 2018). During courtship, one individual attracts an individual of the opposite sex by displaying a repertoire of sensory cues (Preininger et al., 2013; Knörnschild et al., 2014; Ota et al., 2015; Mowles et al., 2017). The multisensory nature of courtship displays across different animal species has been well-summarized thanks to the contributions to this editorial. In humans, vision has been seen as a decisive cue during mate choice, while the other sensory modalities have been largely neglected. Lenschow et al. highlighted that mate selection can be influenced also by tactile and auditory stimuli, such as pleasant social touch and voice pitch. In rodents, olfaction can be considered the mouse counterpart of vision for humans (Luo et al., 2003). During courtship, male rodents couple the release of olfactory signals with the display of ultrasound vocalizations and tactile stimulations (Hoglen et al.). In fruit flies (*Drosophila melanogaster*), males and females engage in stereotyped courtship rituals which include playing songs with extended wings, chasing, tapping, and genital stimulation (Karigo and Deutsch). Across all these examples, multimodal courtship displays help an individual to choose their best mating partner, with every single sensory modality carrying information about the caller that needs to be collected and integrated into the decision-making process for mate selection.

## The neuronal basis of multisensory integration of mating cues

As mating relies on multimodal communication, the ability to encode and integrate sensory information from different sources is essential. In rodents, volatile and non-volatile chemicals activate two parallel olfactory pathways, whose disruption negatively impacts mating success (Hoglen et al.). Mouse courtship USVs selectively recruit neurons of the cochlear nucleus and of the inferior colliculus, where serotonin might already modulate their representation by changing its level depending on female premating choice behavior (Lenschow et al.). Asaba et al. previously showed that the integration of olfactory and acoustic courtship signals plays an important role in male preference (Asaba et al., 2014). They now demonstrated that the prelimbic cortex differentially responds to USVs when they are coupled with sexual odors, suggesting a role of this area in integrating multimodal cues for mate preference (Asaba et al.). Humans also show overlapping circuits with those found in mice for the processing of acoustic and tactile signals, with a strong contribution of higher cortical areas (Lenschow et al.). Importantly, the recruitment of downstream areas activated by sexual cues is conserved across animal species, including regions in the amygdala, hypothalamus, hippocampus, and prefrontal cortex (Hoglen et al.; Karigo and Deutsch; Lenschow et al.).

Sensory integration plays a pivotal role not just during mate choice but also throughout all phases of mating, including copulatory and post-mating behaviors. Martinez-Rivera et al. investigated the role of cutaneous superficial neuromasts in controlling effective sperm transfer in Western mosquitofish (*Gambusia affinis*), showing impairment of gonopodial movements after neuromasts removal. Moreover, neural circuits for sexual cue processing have evolved together with those involved in the generation of courtship displays, with conserved and species-specific neural pathways across animals (Kelley et al.).

## Mating complexity requires flexible neuronal circuits

A fundamental aspect of mating resides in its intrinsic variability due to contextual changes. Karigo and Deutsch provided a comprehensive review of the major factors that influence mating behavior in both mice and flies, including hormonal regulation, social hierarchy, sexual experience, and environmental conditions. As mating needs to adapt to contextual changes, neural circuits governing sexual behaviors require to be flexible. For example, hormonal production changes during development or seasons, with a strong impact on the processing of sexual information (Karigo and Deutsch), and reproductive success (Kelley et al.). The type of relationship established between individuals also influences the characteristics of mating neuronal circuits. Lopez-Gutierrez provided a comparative overview of neural pathways that regulate mating in monogamous species and the role of neuromodulatory factors. A peculiar aspect of monogamous relationships is the need to establish a long-lasting pair bond between the two individuals. Many of the brain regions which have been shown to be involved in bond formation overlap with those recruited during social interaction in non-monogamous species. This suggests a specialization of these areas for the pair bond formation, rather than the emergence of novel pathways dedicated to this process (Lopez-Guiterrez et al.). Finally, the type of established relationship can impact the processing of sensory information. In humans, short-term mating strategies may rely on more immediate sensory signals compared to long-term, which instead may involve extended courtship behavior and contextual sensory cues (Lenschow et al.).

## Conclusions

What emerges from the contributions to this special issue is the central multimodal nature of mating behaviors, where multisensory cues are integrated with external and internal factors to help animals find their best mating partner. Despite these advances in the field, the details of how the brain integrates these multimodal sexual cues in the context of mating are still unclear. Moreover, how these circuits adapt and change based on extrinsic and intrinsic factors, and the extent to which they are conserved in different animal species is largely unknown. Thanks to technical advances in the study of neural circuits, future research will reveal which brain circuits are involved in the control of mating, their flexibility depending on the context, and how much they are evolutionally conserved.

## Author contributions

SZ wrote the first draft of the work and AP and SB revised it. All authors listed have made a substantial, direct, and intellectual contribution to the work. All authors approved it for publication.
